# Circulating Progenitor Cells and Vascular Dysfunction in Chronic Obstructive Pulmonary Disease

**DOI:** 10.1371/journal.pone.0106163

**Published:** 2014-08-29

**Authors:** Sandra Pizarro, Jéssica García-Lucio, Víctor I. Peinado, Olga Tura-Ceide, Marta Díez, Isabel Blanco, Marta Sitges, Jordi Petriz, Yolanda Torralba, Pedro Marín, Josep Roca, Joan Albert Barberà

**Affiliations:** 1 Department of Pulmonary Medicine, Hospital Clinic-Institut d’Investigacions Biomèdiques August Pi i Sunyer (IDIBAPS), University of Barcelona, Barcelona, Spain; 2 Department of Cardiology, Hospital Clinic-Institut d’Investigacions Biomèdiques August Pi i Sunyer (IDIBAPS), University of Barcelona, Barcelona, Spain; 3 Department of Cryopreservervation, Hospital Clínic-Institut d’Investigacions Biomèdiques August Pi i Sunyer (IDIBAPS), University of Barcelona, Barcelona, Spain; 4 Centro de Investigación Biomédica en Red de Enfermedades Respiratorias (CIBERES), Madrid, Spain; 5 Department of Cytometry, Institut de Recerca, Hospital Universitari Vall d’Hebron, Barcelona, Spain; Vanderbilt University Medical Center, United States of America

## Abstract

**Background:**

In chronic obstructive pulmonary disease (COPD), decreased progenitor cells and impairment of systemic vascular function have been suggested to confer higher cardiovascular risk. The origin of these changes and their relationship with alterations in the pulmonary circulation are unknown.

**Objectives:**

To investigate whether changes in the number of circulating hematopoietic progenitor cells are associated with pulmonary hypertension or changes in endothelial function.

**Methods:**

62 COPD patients and 35 controls (18 non-smokers and 17 smokers) without cardiovascular risk factors other than cigarette smoking were studied. The number of circulating progenitors was measured as CD45^+^CD34^+^CD133^+^ labeled cells by flow cytometry. Endothelial function was assessed by flow-mediated dilation. Markers of inflammation and angiogenesis were also measured in all subjects.

**Results:**

Compared with controls, the number of circulating progenitor cells was reduced in COPD patients. Progenitor cells did not differ between control smokers and non-smokers. COPD patients with pulmonary hypertension showed greater number of progenitor cells than those without pulmonary hypertension. Systemic endothelial function was worse in both control smokers and COPD patients. Interleukin-6, fibrinogen, high sensitivity C-reactive protein, vascular endothelial growth factor and tumor necrosis factor were increased in COPD. In COPD patients, the number of circulating progenitor cells was inversely related to the flow-mediated dilation of systemic arteries.

**Conclusions:**

Pulmonary and systemic vascular impairment in COPD is associated with cigarette smoking but not with the reduced number of circulating hematopoietic progenitors. The latter appears to be a consequence of the disease itself not related to smoking habit.

## Introduction

Chronic obstructive pulmonary disease (COPD) is associated with alterations in pulmonary vessel structure and function, which frequently result in pulmonary hypertension (PH), a major factor associated with reduced survival [1]. Endothelial cell damage and dysfunction produced by cigarette smoke or inflammatory mediators appear to be at the origin of the remodeling process of pulmonary vasculature and, hence, of PH [2]. The mechanisms involved in pulmonary vascular remodeling are not fully understood. Intimal hyperplasia, the most prominent feature of pulmonary vascular remodeling, is produced by the proliferation of poorly differentiated smooth muscle cells (SMCs) and the deposition of collagen and elastic fibers [3]. The origin of these cells and the causes of their uncontrolled proliferation are uncertain.

Circulating hematopoietic progenitor cells conform a population of progenitor cells originated in the bone marrow with the ability to be mobilized in response to vascular injury. These cells play a key role in tissue repair and participate in the progression of pre-existing lesions [4]; [5]. We have previously shown an increased number of CD133^+^ progenitor cells within the wall of pulmonary arteries of COPD patients [6]. CD133^+^ cells showed the capacity to migrate from vessel lumen into the intima and differentiate into SMCs [7]. This finding suggested that circulating bone marrow-derived progenitor cells might be involved in pulmonary vessel remodeling.

Patients with cardiovascular risk factors may show reduced numbers of circulating progenitors and impaired endothelial function in systemic arteries [8]; [9]. Both changes are associated with shorter survival and increased number of cardiovascular events [8]; [10]; [11].

Cardiovascular events are common in COPD patients. Recent studies imply that COPD patients have lower circulating progenitors [12]–[14] and dysfunctional systemic arteries [15]–[20]. This reduction of circulating progenitor cells in COPD patients might lead to a lower repair capacity and consequently to an altered vascular function [21]–[23]. Little is known about the origin of these changes in COPD nor if they are associated with alterations in the pulmonary circulation which may in turn progress to PH, a factor also associated with poor prognosis [1] .

We hypothesized that in COPD, the alterations in pulmonary circulation are associated with changes in the number of circulating progenitors by mechanisms similar to those involved in the systemic circulation and also with changes in systemic vessel function. Thereby, suggesting that similar mechanisms of vascular impairment could occur in both systemic and pulmonary circulations.

Accordingly, the present study aimed to investigate whether COPD patients, without any cardiovascular risk factor other than cigarette smoking, have different number of circulating hematopoietic progenitor cells. In addition, we aimed to study whether this was associated with an altered endothelial function of systemic arteries and/or with the presence of PH. Sex and age-matched healthy subjects, non-smokers and current smokers, were used as control groups.

## Methods

Sixty-two COPD patients and 35 healthy controls (18 non-smokers and 17 current smokers), aged between 50–75 years, were enrolled in the study. Patients were recruited from the outpatient clinic. The study was approved by the internal review board and all subjects gave written informed consent. All subjects were free of cardiovascular risk factors except cigarette smoking. Patients with established cardiovascular, cerebral-vascular and/or metabolic diseases, subjects under vasodilator, anti-lipidemic or anti-diabetic therapy were excluded.

COPD was diagnosed according to current guidelines [24]. Patients were clinically stable at the time of the study without exacerbation episodes or oral steroid treatment for the previous 4 months. All patients were on regular bronchodilator treatment and most of them received inhaled corticosteroids. No patient was receiving systemic steroids. Patients treated with long-term oxygen were included. In control subjects, the absence of lung disease was confirmed by clinical evaluation, chest radiograph and lung function tests.

### Measurements

All subjects underwent standard evaluation by means of medical history, clinical examination, lung function tests, arterial blood gases and electrocardiogram. COPD patients underwent additional Doppler echocardiography for the assessment of associated PH. PH was considered when tricuspid regurgitant peak velocity was >2.8 m/s [25], which is equivalent to a trans-tricuspid systolic pressure gradient >31 mmHg. Three patients had PH confirmed by right heart catheterization.

Interleukin-6 (IL-6), fibrinogen, C-reactive protein (hsCRP), endothelin-1, nitrites/nitrates, vascular endothelial growth factor (VEGF), angiopoietin-2 and brain natriuretic peptide (BNP) were measured. In 31 COPD patients and 25 control subjects, tumor necrosis factor-alpha (TNF-α) was additionally measured (see Data S1).

### Circulating progenitor cells

The number of circulating hematopoietic progenitors was evaluated by flow cytometry using antibodies against CD45 (pan-leukocyte marker), CD133 (sub-population of hematopoietic stem cells) and CD34 (mature and progenitor endothelial cells) as previously described [26]. In brief, mononuclear cells (MNCs) were isolated by Ficoll density gradient separation, washed once with phosphate buffered saline (PBS) supplemented with 2% fetal calf serum (FCS) and ressuspended at 2×10^6^ cells (control tube) and at 4×10^6^ cells (sample tube). MNCs were stained and analyzed for phenotypic expression of surface markers using pre-conjugated anti-human monoclonal antibodies; anti-CD45-FITC, anti-CD34-PECy7 and anti-CD133-PE. The fluorescence minus one technique [27] was employed to provide negative controls. After 45 minutes of incubation, cells were washed, ressuspended into 500 ul of PBS+2% FCS and proceeded to flow-cytometric analysis. 80,000 events were gated in the lymphocyte region (Figure S1).

### Endothelial function

Endothelial function was assessed by high resolution ultrasound as the change in brachial artery diameter in response to reactive hyperemia (flow-mediated dilation) [28]. Endothelium-independent, nitroglycerine-mediated dilation was also measured. Additional details are provided in Data S1.

### Statistical Analysis

Data are expressed as mean±SD for normally distributed data or as median with interquartile range for skewed distributions. Group comparisons were performed using one way ANOVA and post hoc pairwise comparisons using the Student Newman-Keuls test for normally distributed variables or the Kruskal-Wallis and the Dunn’s test for non-normally distributed variables. Correlations between variables were analyzed with Pearson’s or Spearman’s coefficient depending on data distribution. A p value<0.05 was considered statistically significant.

## Results

Anthropometric, clinical and functional characteristics of patients and control subjects are shown in Table 1. Groups were well matched with respect to age, body mass and smoking status. Patients with COPD had moderate to severe airflow limitation. Thirty-seven of them (60%) were in spirometric GOLD [24] stage II, 13 (21%) in stage III, and 12 (19%) in stage IV. They showed air trapping, mild to moderate hypoxemia and PaCO_2_ within the normal range. Half of the COPD patients and all control smokers were current smokers. Eight patients were receiving long-term oxygen therapy. There were no differences in the Framingham risk score between control subjects and COPD patients. Echocardiogram was performed in 57 patients with COPD. Twenty of them had suspected PH as previously defined and 37 did not.

**Table 1:** Clinical characteristics, lung function, cardiovascular and laboratory measurements.

	Control nonsmokers(n = 18)	Control Smokers(n = 17)	COPD Patients(n = 62)
Age, years	58±6	59±8	62±8
Male sex, n (%)	7 (39%)	12 (71%)	58 (94%)*
Body mass index (Kg/m^2^)	25±3	25±3	26±3
Framingham risk score‡	6±3	9±5	12±6*
Current smokers, %	0	100*	47*
Smoking history, pack-years	0	41±21*	60±32* †
Expiratory carbon monoxide, ppm	0.9±0.6	2.9±1.7*	2.5±2.4*
FEV_1_, % predicted	106±7	100±12	53±18* †
FEV_1_/FVC	82±5	78±5	48±13* †
TLC, % predicted	98±8	102±16	109±16*
RV, % predicted	102±18	112±18	168±51* †
DL_CO_, % predicted	91±11	79±14	61±19* †
PaO_2_, mmHg	97±8	86±10	72±8* †
PaCO_2_, mmHg	38±4	35±3	38±4†
Systolic blood pressure, mmHg	115±12	113±9	120±13
Diastolic blood pressure, mmHg	73±10	70±6	72±9
Total Cholesterol, mg/dL	227±34	195±33*	207±36*
Triglycerides, mg/dL	93±41	96±32	111±48
HDL, mg/dL	61±20	49±16	53±14
LDL mg/dL	148±24	127±30	132±30
Glucose mg/dL	93±9	89±13	93±20
Leukocyte count, ×10^9^/L	5.42±0.86	8.12±1.52*	8.10±1.82*
Lymphocyte count, ×10^9^/L	1.57±0.43	2.07±0.69*	2.03±0.65*
Monocyte count, ×10^9^/L	0.25±0.067	0.42±0.15*	0.46±0.14*
Neutrophils, ×10^9^/L	3.29±0.66	5.25±1.60*	5.23±1.52*
Red blood cells, ×10^12^/L	4.74±0.50	4.64±0.40	4.79±0.37
Hemoglobin (g/L)	143±13	145±17	149±12
Platelet count, ×10^9^/L	251±46	290±97	254±61

Data are shown as mean ± SD.

Definition of abbreviations: COPD: Chronic obstructive pulmonary disease; FEV_1_: post-bronchodilator forced expiratory volume in the first second; TLC: total lung capacity; RV: residual volume; DLco: diffusing capacity of the lung for carbon monoxide; PaO_2_: partial pressure of arterial oxygen; PaCO_2_: partial pressure of arterial carbon dioxide; HDL: high-density lipoprotein; LDL: low-density lipoprotein.

*P<0.05 compared with control nonsmokers.

† P<0.05 compared with control smokers.

‡ The Framingham risk score can range from −6 to 19, with higher scores indicating greater cardiovascular risk.

White blood cell counts were in the normal range in all subjects. Yet, COPD patients and control smokers showed approximately 1.5 fold higher blood leukocyte, lymphocyte, neutrophil and monocyte cell counts compared with control non-smokers. Red blood cells and platelet counts were similar between groups. Hemoglobin concentration was within the normal range in all subjects (Table 1).

### Circulating hematopoietic progenitor cells

**Figure 1:**
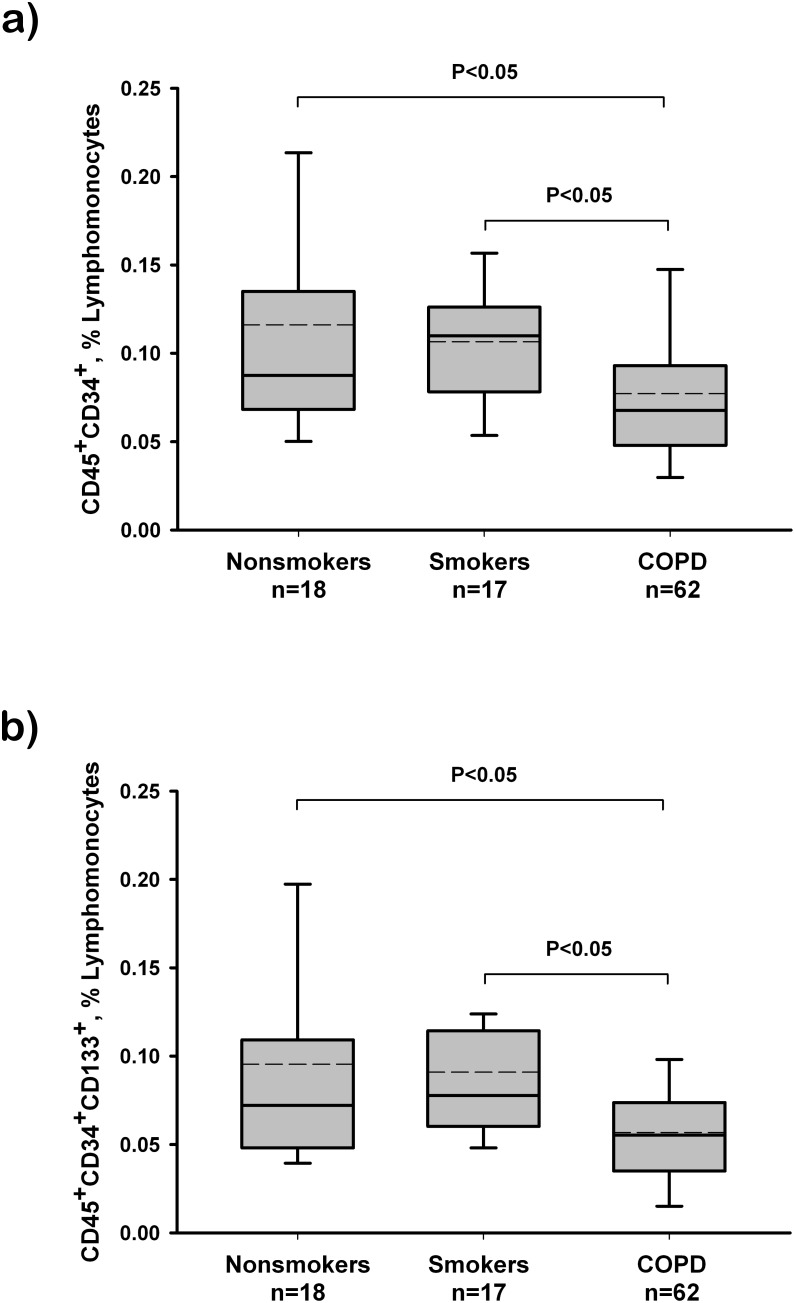
Number of circulating hematopoietic progenitors cells in nonsmokers, control smokers and COPD patients. (A) Number of CD45+CD34+ labelled cells, expressed as percent of lymphomonocytes. (B) Number of CD45+CD34+CD133+ labelled cells, expressed as percent of lymphomonocytes. The box represents the interquartile range. The solid line indicates the median and the dashed line indicates the mean. The whiskers extend from the box to the 90th and 10th percentiles. One-way analysis of variance and post hoc pairwise comparisons using the Dunn’s test.

Circulating hematopoietic progenitor cells were defined as CD45^+^CD34^+^ or CD45^+^CD34^+^CD133^+^ cells. The number of circulating progenitors corrected by the total number of leukocytes was highly variable among individuals, particularly in control subjects. The number of CD45^+^CD34^+^ cells were significantly reduced in COPD patients compared with both smokers and non-smokers control groups (Figure 1A). Similarly, CD45^+^CD34^+^CD133^+^ labeled cells were significantly reduced in COPD patients as compared with both control groups (Figure 1B). The number of CD45^+^CD133^+^ labeled cells did not show any significant difference between groups (data not shown). No differences were seen in the number of CD45^+^CD34^+^ or CD45^+^CD34^+^CD133^+^ between the non-smokers and the smokers control groups.

**Figure 2:**
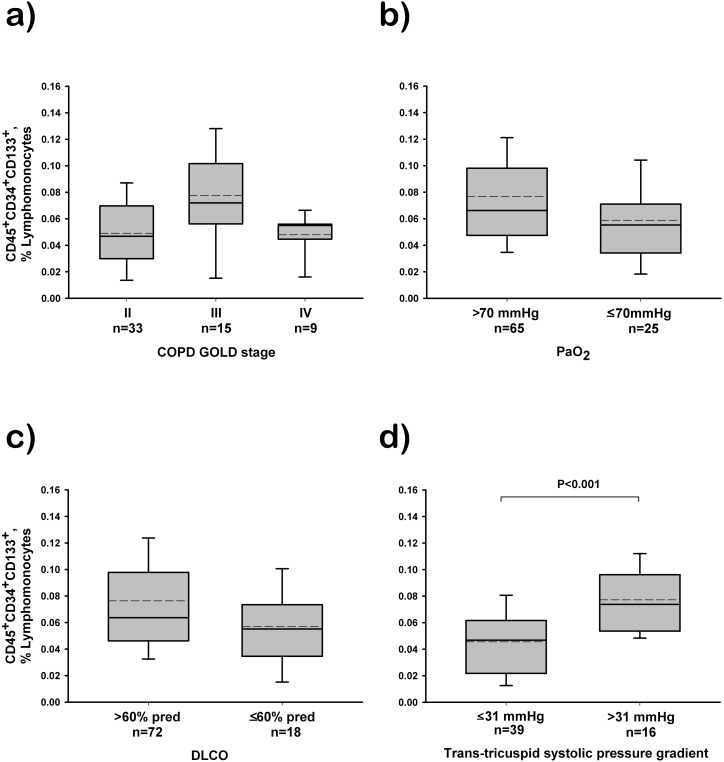
Relationship between the number of circulating CD45+CD34+CD133+ labelled cells and COPD severity. (A) Patients grouped according to the spirometric GOLD stage. (B) Patients grouped according to PaO_2_ value above or below the median value (70 mmHg). (C) Patients grouped according to DLCO above or below the median value (60% predicted). (D) Patients grouped according to trans-tricuspid systolic pressure gradient suggestive of pulmonary hypertension (>31 mmHg), assessed by Doppler echocardiography. The box represents the interquartile range. The solid line indicates the median and the dashed line indicates the mean. The whiskers extend from the box to the 90th and 10th percentiles. One-way analysis of variance post hoc pairwise comparisons using the Kruskal-Wallis and the Dunn’s test.

The number of circulating CD45^+^CD34^+^CD133^+^ progenitors in COPD patients was not related to the disease severity, as there were no differences among patients with moderate, severe and very severe COPD according to the spirometric GOLD stage (Figure 2A). Furthermore, we did not find differences in the number of circulating hematopoietic progenitors when comparing patients with PaO_2_ or DLco above and below the median value (Figure 2B, C). By contrast, COPD patients with increased trans-tricuspid systolic pressure gradient suggestive of PH showed an increased number of circulating progenitors compared with those with normal gradient (Figure 2D).

Within the COPD group there were both current smokers and ex-smokers. To evaluate whether smoking status could influence circulating progenitor counts, group comparisons were repeated excluding those COPD patients who were current smokers. The re-analysis showed that COPD patients had lower levels of circulating progenitors than controls (non-smokers and smokers) irrespective of the smoking status (data not shown).

### Endothelial function of systemic arteries

Baseline brachial artery diameter was greater in both COPD patients and control smokers compared with non-smokers (Table 2). Flow-mediated dilation of the brachial artery was highly variable among individuals. Whereas the majority of non-smokers showed a vasodilator response, both healthy smokers and COPD patients had poor vasodilator responses and in a number of cases a negative change (vasoconstrictor response). Compared with non-smokers, flow-mediated dilation was equally significantly reduced in both the COPD patients and the control smokers (Table 2). To account for the differences in baseline vessel diameter between groups, we calculated the ratio between flow-mediated dilation and baseline brachial artery diameter, which is an index of impaired vascular reactivity independent of vessel diameter [29]. After this correction, both COPD patients and control smokers continued to have lesser flow-mediated dilation compared with non-smokers (Table 2).

**Table 2:** Vascular reactivity of the brachial artery in the study population.

	Control Nonsmokers(n = 18)	Control Smokers(n = 17)	COPD Patients(n = 62)
Baseline brachial artery diameter, mm	4.0 (3.5 to 4.3)	4.4 (3.7 to 4.9)	4.5 (4.3 to 5.2)*
Flow-mediated dilation, % changefrom baseline diameter	2.4 (1.1 to 4.1)	0.0 (−0.8 to 1.6)*	0.9 (−1.3 to 2.3)*
Flow-mediated dilation/Baselinebrachial artery diameter, %/mm	0.60 (0.30 to 1.10)	0.00 (−0.17 to 0.35)*	0.22 (−0.27 to 0.49)*
Nitroglycerine-mediated dilation, %	17.7 (15.3 to 20.6)	15.3 (13.4 to 21.5)	14.3 (9.7 to 19.8)
Hyperemia, % fraction of maximumflow rate between the post-reactivehyperemia and the basal rate,normalized by heart rate	440 (348 to 572)	392 (315 to 611)	431 (369 to 571)

Data are shown as median (interquartile range).

*P<0.05 compared with control nonsmokers.

**Figure 3:**
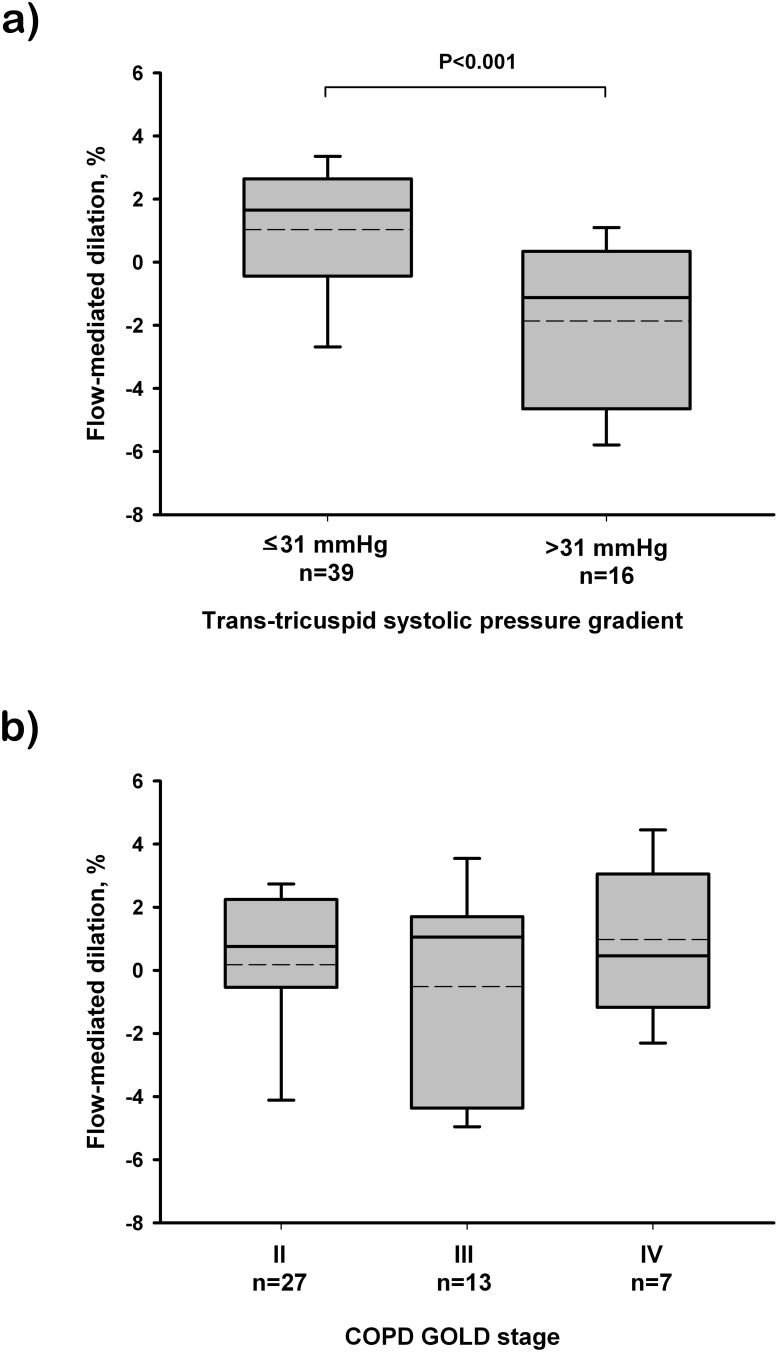
Relationship between endothelial function, assessed by flow-mediated dilation, of the brachial artery and COPD severity. (A) Patients grouped according to trans-tricuspid systolic pressure gradient suggestive of pulmonary hypertension (>31 mmHg), assessed by Doppler echocardiography. (B) Patients grouped according to the spirometric GOLD stage. The box represents the interquartile range. The solid line indicates the median and the dashed line indicates the mean. The whiskers extend from the box to the 90th and 10th percentiles. One-way analysis of variance and post hoc pairwise comparisons using the Dunn’s test.

Among COPD patients, those with suspected PH had lower endothelium-dependent vasodilation than those without PH (Figure 3A). In contrast, there were no differences in flow-mediated dilation between COPD patients grouped according to the spirometric GOLD stage (Figure 3B). Nitroglycerine administration produced significant dilation of the brachial artery in all subjects without significant differences between groups, although it was slightly lower in the COPD group (P = 0.06) (Table 2).

### Inflammatory and vascular markers

Compared with control non-smokers, COPD patients had higher plasma levels of fibrinogen and VEGF, as well as higher serum levels of IL-6, TNF-α and hsCRP. Control smokers had higher levels of angiopoietin-2, fibrinogen, TNF-α and IL-6 than non-smokers, without differences with COPD patients (Table 3).

**Table 3:** Vascular and systemic inflammatory markers in the study population.

	Control Nonsmokers(n = 18)	Control Smokers(n = 17)	COPD patients(n = 62)
IL-6, % detectable*	11	23	43
IL-6, pg/mL	35 (5 to 65)	6 (5 to 19)	8 (5 to 22)
VEGF, % detectable*	28	40	65
VEGF, pg/mL	38 (20 to 75)	43 (20 to 102)	60 (35 to 100)
hsCRP, mg/dL	0.12 (0.08 to 0.19)	0.24 (0.10 to 1.44)	0.45 (0.19 to 0.92)†
BNP, pg/mL	17.5 (9.6 to 20.6)	20.5 (13.0 to 25.2)	21.9 (8.4 to 29.4)
Fibrinogen, g/L	3.3 (2.8 to 3.8)	3.9 (3.3 to 4.2)†	4 (3.6 to 4.9)†
Nitrites/nitrates, nMol/mL	17.9 (13.9 to 27.3)	21.4 (19.4 to 27.8)	22.3 (15.2 to 32.5)
Endothelin-1, pmol/L	5.7 (4.6 to 7.4)	5.7 (4.4 to 6.4)	5.1 (4.0 to 7.0)
Angiopoietin-2, pg/mL	408 (263 to 486)	434 (390 to 649)†	406 (333 to 528)
TNF-α, pg/mL‡	4.0 (3 to 5)	5.5 (5 to 6)	6.0 (5 to 7)†

Data are shown as median (interquartile range).

Definition of abbreviations: IL-6: Interleukin-6; VEGF: vascular endothelial growth factor; hsCRP: high sensitive C-reactive protein; BNP: Brain natriuretic peptide; TNF-α: tumour necrosis factor α.

*p<0.05 Chi-square.

† P<0.05 compared with control nonsmokers.

‡ TNF-α was measured in 23 control subjects (13 nonsmokers and 10 smokers) and 30 COPD patients.

No differences in the serum or plasma levels of the different biomarkers were found in COPD patients according to the severity of airflow obstruction or the presence/absence of PH.

### Relationship among circulating progenitor cells, systemic endothelial function, lung function and vascular biomarkers

**Figure 4:**
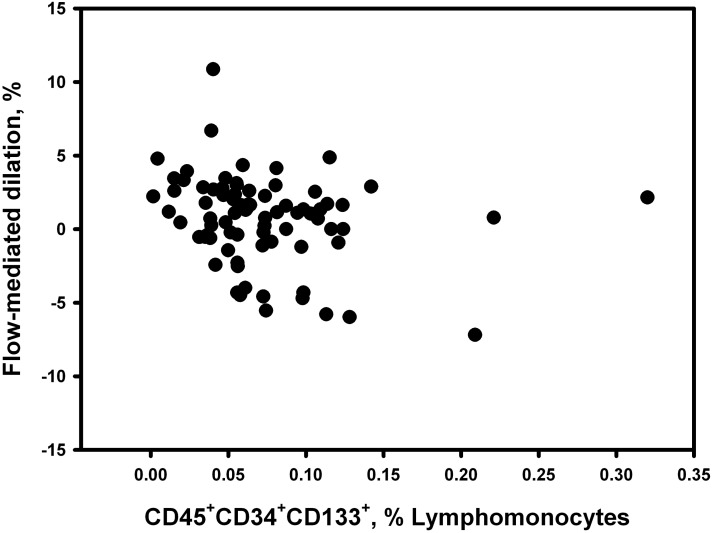
Inverse correlation between progenitor cells and endothelial function. Inverse relationship between the number of circulating CD45+CD34+CD133+ progenitor cells and the endothelial function, assessed by flow-mediated dilation, of the brachial artery in patients with COPD (r = −0.27, P<0.05).

In the entire study population, we observed statistically significant correlations between the number of circulating progenitor cells and FEV_1_, DLco and PaO_2_ (r = 0.21, 0.23 and 0.28, respectively, P<0.05 each) consistent with the differences between groups. We did not observe any significant correlation between the number of circulating progenitors and flow-mediated dilation in the whole series. However, within the COPD group the number of circulating progenitors was inversely related to the flow-mediated dilation of the brachial artery (Figure 4). Patients with worse endothelial function had greater numbers of circulating progenitors. The levels of inflammatory and vascular biomarkers were not related either to circulating progenitors or systemic endothelial function both in the entire study population and in the COPD group.

## Discussion

Results of the present study show that patients with COPD, without cardiovascular comorbidities and risk factors other than cigarette smoking, have reduced numbers of CD45^+^CD34^+^CD133^+^ circulating progenitor cells compared with both smokers and non-smokers control groups and an impaired endothelial function in systemic arteries. Among COPD patients the highest numbers of circulating hematopoietic progenitor cells were observed in those who had poorer systemic endothelial function and increased pulmonary arterial pressure. Healthy smokers also showed systemic endothelial dysfunction, but in contrast to COPD patients the number of circulating progenitors did no differ from non-smokers.

In COPD, previous studies have investigated the number of endothelial progenitor cells [12]; [14]; [30]–[32]. While most of the studies indicate lower levels of circulating progenitor cells in COPD patients compared with controls, others do not [30]. This discrepancy between results most probably is due to significant methodological differences between laboratories and failure to reliably match for cardiovascular risk factors, gender and age [30]. In this study, we aimed to exclude subjects with cardiovascular risk factors and separate healthy non-smokers from healthy smokers. These grouping criteria are significant since it has been shown that these factors are associated with a reduction of circulating progenitors and could provide false positive results [8]; [9].

It is of note that all COPD patients were current or former smokers. Nevertheless, smoking status did not seem to account for the reduction in circulating progenitors since smokers without COPD had greater number of progenitor cells than in COPD patients and similar to those observed in non-smokers. The effect of cigarette smoking on progenitor cells is controversial. While some studies have reported a significant increase when incubated *in vitro* with nicotine [33] or increased circulating number after second hand smoke exposure [34], others have reported reduced circulating progenitor cells in healthy smokers [35]. Our observation of similar numbers of circulating progenitors in healthy smokers and in non-smokers suggests that in the absence of other cardiovascular risk factors (namely diabetes, hypercholesterolemia or systemic arterial hypertension) cigarette smoking does not seem to alter them. Accordingly, our results indicate that in COPD the reduction in circulating progenitor cells is independent of the smoking habit and appears to be related to the disease itself.

The mechanisms by which circulating hematopoietic progenitor cells may be reduced in COPD are not apparent. In contrast to previous results [12]; [14], in our study the number of circulating hematopoietic progenitor cells was not related to disease severity, as assessed either by spirometric GOLD staging or by conventional lung function measurements (FEV_1_, DLco, PaO_2_). Only the presence of suspected PH was associated with a slight increase in CD45^+^CD34^+^CD133^+^ cells. Several hypotheses have been suggested to explain the reduction of progenitor cells in COPD: impairment of the bone marrow to produce and release progenitor cells [14], recruitment of circulating progenitors in injured lung vessels [6]; [36]–[38], increased apoptosis [12], and the effect of inflammatory or angiogenic mediators [39]. In our series, COPD patients had normal peripheral blood cell counts. Even though, white blood cell counts were higher in the COPD group than in control non-smokers. Accordingly, bone marrow impairment does not seem to account for the observed reduction in circulating progenitors. We did not test for the presence of apoptosis, thus we cannot rule out the suggestion that the decrease of circulating progenitor cells could result from increased cell death rate due to apoptosis [12]. In a similar way, we cannot disregard that circulating progenitor cells could be recruited in injured vessels. In fact, our group has previously demonstrated the presence of cells expressing CD45 and CD133 markers in the wall of pulmonary arteries in COPD patients [6], potentially suggesting recruitment of bone marrow-derived progenitor cells that might eventually contribute to an ongoing process of endothelium repair or be involved in the pathogenesis of vessel remodelling [6]. Fadini *et al* [12], reported an inverse relationship between PaO_2_ and circulating progenitor cells, suggesting that hypoxia could stimulate progenitor cell mobilization from the bone marrow and their localization at the damaged pulmonary endothelium. In our study we did not observe any relationship between the number of circulating progenitors and PaO_2_. Yet, patients with suspected PH showed greater numbers of circulating progenitor cells than those with systolic PAP within the normal range [25], a finding that would be in favor that damaged pulmonary endothelium could stimulate the mobilization of progenitor cells from the bone marrow.

The potential role of inflammatory and angiogenic mediators in modulating progenitor cell numbers cannot be disregarded. Furthermore, levels of cytokines such as VEGF can differ according to tissue location and disease stage [40]; [41]. Increased levels of VEGF are associated with an increased production and mobilization of progenitor cells from the bone marrow [39], whereas TNF-α may impair its production [42]; [43]. In this study, both plasma levels of VEGF and TNF-α were increased in the COPD group, as compared with the non-smokers, as in previous studies [14]; [44]. However, the enhanced levels of VEGF did not correlate with the increased numbers of circulating progenitor cells.

The concurrence of reduced numbers of circulating progenitor cells and elevated plasma levels of VEGF and TNF-α have also been reported in other conditions where systemic inflammation may contribute to vascular impairment, as in rheumatoid arthritis [39] or congestive heart failure [45]. Interestingly, treatment with TNF-α blockers increases the number of circulating progenitor cells and enhances their endothelial differentiation [39]. Accordingly, there may be a connection between systemic inflammation and CD45^+^CD34^+^CD133^+^ count reduction in COPD, where TNF-α might play a pivotal role [43].

Whatever the mechanism accounting for circulating progenitor cell reduction in COPD, it is interesting to note that in our series COPD patients with PH had greater numbers of circulating progenitor cells than those without suspected PH. This might suggest that these patients may preserve to some extent the bone marrow capacity to mobilize these cells in response to lung vascular injury.

Both COPD patients and healthy smokers showed impaired endothelial function when compared with non-smokers, in agreement with previous studies [15]; [16]; [20]. COPD patients also showed a trend towards reduced endothelium-independent vasodilation, suggesting that vascular abnormalities in COPD may not be restricted to the endothelium and affect also smooth muscle cells [20]. Furthermore, the baseline brachial artery diameter was greater in healthy smokers and in COPD patients. Differences in vessel size could be explained by fibrous remodeling of the media in large arteries that may result in vessel stiffness and dilatation [20].

Endothelial function of systemic arteries was inversely related to the number of circulating progenitor cells in COPD patients, being the patients with lesser flow-mediated dilation those with greater number of circulating progenitor cells. This finding agrees with the observation of greater CD45^+^CD34^+^CD133^+^ numbers in COPD patients with suspected PH, suggesting that vascular damage, either in the pulmonary or the systemic circulation, might stimulate the bone marrow and promote the release of progenitor cells. This data contrasts with what has been observed in healthy individuals with different degrees of cardiovascular risk, where endothelial function was directly related to the number of circulating progenitors [8].

Interestingly, healthy smokers showed similar endothelial dysfunction in the brachial artery than COPD patients, but the number of circulating CD45^+^CD34^+^CD133^+^ was significantly increased compared to COPD patients and unrelated to the endothelial function. This suggests that endothelial dysfunction and changes in progenitor cell numbers may follow different pathways in COPD. Presumably, endothelial dysfunction results from the effect of cigarette smoke products on endothelial cells, likely as a result of oxidative stress [46], whereas the impaired release of bone marrow-derived progenitor cells may result from mechanisms not linked to cigarette consumption and likely related to changes induced by the disease itself.

In the present series, COPD patients had increased levels of IL-6, hsCRP, VEGF, TNF-α and fibrinogen, compared with non-smokers. Healthy smokers also had higher levels of IL-6, fibrinogen, angiopoietin-2 and TNF-α than non-smokers. No correlation was found between the number of circulating CD45^+^CD34^+^CD133^+^, systemic endothelial function and any of these biomarkers. This is consistent with recent studies [14]; [20]; [44] and denotes the complex interactions between markers of systemic inflammation and vascular impairment.

This study has some limitations. Firstly, the study population was carefully selected to avoid any cardiovascular risk factor other than cigarette smoking. This is not representative of the common COPD population since these patients usually present additional factors for cardiovascular risk. Nevertheless, the absence of cardiovascular risk factors allowed us to avoid potential confounding factors on the pathogenesis of endothelial dysfunction and impaired release of progenitors in COPD. Secondly, we did not investigate the dynamics of circulating progenitor cells and thus we cannot report on whether observed differences reflect alterations in progenitor cell mobilization, survival or tissue uptake. Thirdly, events in systemic circulation such as reduced levels of progenitor cells may not necessarily reflect changes in the lung tissue. Finally, PH was assessed by echocardiography in most patients and only a few patients had PH diagnosed by right heart catheterization. Despite we took into account recent recommendations on the predictive diagnostic value of echocardiography for PH [25], we cannot discard that some of our cases with suspected PH would be false positives or in the other hand, patients without suspected PH would be false negatives. Given the purposes of the present investigation, it was considered not appropriate to perform right heart catheterization to subclassify COPD patients.

In summary, the present study shows that circulating hematopoietic progenitor cells are reduced in COPD which seems to be independent of the smoking habit. This suggests that lower number of progenitor cells is probably caused by factors related to the disease itself, presumably systemic inflammation. Yet, COPD patients have preserved capacity to mobilize progenitor cells, as suggested by the observation that those with greater vascular damage, either at the pulmonary or the systemic circulation, have higher numbers of circulating CD45^+^CD34^+^CD133^+^ cells. We have also shown that cigarette smoking plays a key role in endothelial dysfunction, which is present in both healthy smokers and COPD patients. The association of endothelial dysfunction and impaired repair capacity, as a consequence of reduced progenitor cell mobilization, may contribute to the cardiovascular comorbidity frequently shown in COPD, especially if additional risk factors concur.

## Supporting Information

Figure S1 (TIF)

Data S1 (DOC)
