# Correction: Circulating Progenitor Cells and Vascular Dysfunction in Chronic Obstructive Pulmonary Disease

**DOI:** 10.1371/journal.pone.0115566

**Published:** 2014-12-05

**Authors:** The PLOS ONE Staff

Figures 2 and 4 are incorrect. There is also an error in the legend for Figure 4, “Inverse correlation between progenitor cells and endothelial function.” The authors have provided a corrected version of Figure 2, Figure 4, and the Figure 4 legend here.

**Figure 2:**
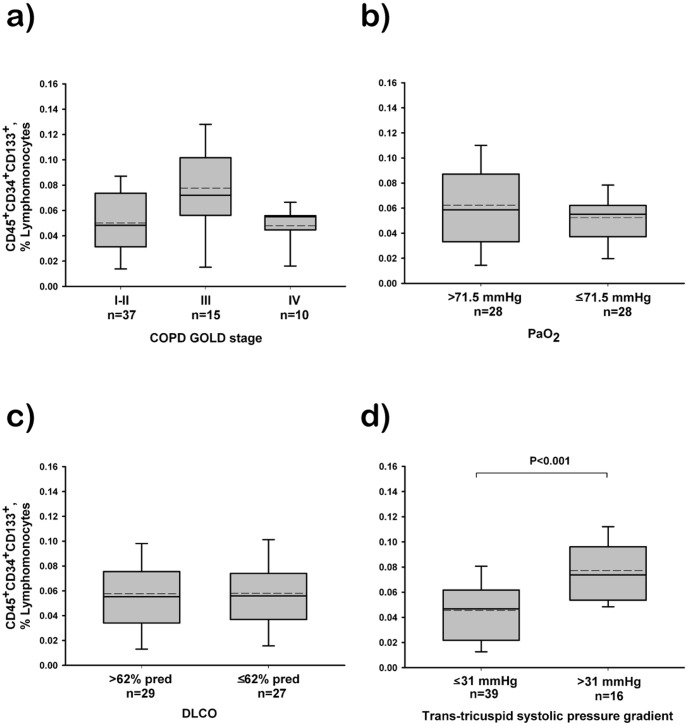
Relationship between the number of circulating CD45+CD34+CD133+ labelled cells and COPD severity. (A) Patients grouped according to the spirometric GOLD stage. (B) Patients grouped according to PaO2 value above or below the median value (70 mmHg). (C) Patients grouped according to DLCO above or below the median value (60% predicted). (D) Patients grouped according to trans-tricuspid systolic pressure gradient suggestive of pulmonary hypertension (>31 mmHg), assessed by Doppler echocardiography. The box represents the interquartile range. The solid line indicates the median and the dashed line indicates the mean. The whiskers extend from the box to the 90th and 10th percentiles. One-way analysis of variance post hoc pairwise comparisons using the Kruskal-Wallis and the Dunn’s test.

**Figure 4:**
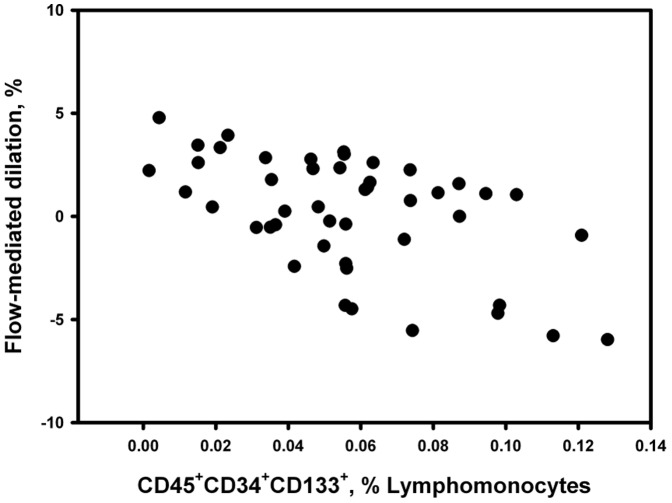
Inverse correlation between progenitor cells and endothelial function. Inverse relationship between the number of circulating CD45+CD34+CD133+ progenitor cells and the endothelial function, assessed by flow-mediated dilation, of the brachial artery in patients with COPD (r =  –0.49, P<0.001).

## References

[1] Pizarro S, García-Lucio J, Peinado VI, Tura-Ceide O, Díez M, et al (2014) Circulating Progenitor Cells and Vascular Dysfunction in Chronic Obstructive Pulmonary Disease. PLoS ONE 9(8): e106163 doi:10.1371/journal.pone.0106163 25171153 10.1371/journal.pone.0106163 PMC4149524

