# Supplementary figures and images for: Circulating Progenitor Cells and Vascular Dysfunction in Chronic Obstructive Pulmonary Disease
Source: PLoS One. 2014 Aug 29;9(8):e106163. doi: 10.1371/journal.pone.0106163 (PMC4149524; doi:10.1371/journal.pone.0106163)

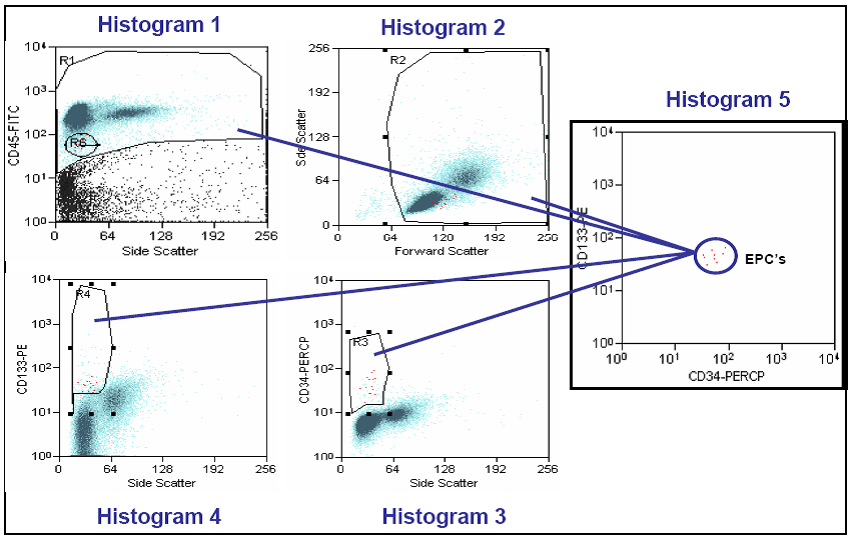

Supplement: Figure S1 — (TIF) [file pone.0106163.s001.tif]
