# Supplementary material for: Circulating Progenitor Cells and Vascular Dysfunction in Chronic Obstructive Pulmonary Disease
Source: PLoS One. 2014 Aug 29;9(8):e106163. doi: 10.1371/journal.pone.0106163 (PMC4149524; doi:10.1371/journal.pone.0106163)
Supplement: Data S1 — (DOC) [file pone.0106163.s002.doc]

**Online Data Supplement**

**CIRCULATING PROGENITOR CELLS AND VASCULAR DYSFUNCTION IN CHRONIC OBSTRUCTIVE PULMONARY DISEASE**

Sandra Pizarro^1^, Jéssica García-Lucio^1^, Víctor I. Peinado^1,4^, Olga Tura-Ceide^1,4^, Marta Díez^1^, Isabel Blanco^1,4^, Marta Sitges^2^, Jordi Petriz^5^, Yolanda Torralba^1,4^, Pedro Marín^3^, Josep Roca^1,4^, and Joan Albert Barberà^1,4^.

Departments of ^1^Pulmonary Medicine, ^2^Cardiology and ^3^Cryopreservervation; Hospital Clínic-Institut d’Investigacions Biomèdiques August Pi i Sunyer (IDIBAPS); University of Barcelona. ^4^Centro de Investigación Biomédica en Red de Enfermedades Respiratorias (CIBERES); Spain. ^5^Department of Cytometry, Institut de Recerca, Hospital Universitari Vall d'Hebron, Barcelona, Spain.

**SUPPLEMENTAL METHODS**

**Circulating progenitor cells**

Venous blood samples were drawn from study subjects and transferred to vacutainers with EDTA K3, maintaining sterile conditions. Blood was mixed with phosphate buffered saline (PBS) with 2% fetal calf serum (FCS). Peripheral blood mononuclear cells were obtained by standard Ficoll gradient centrifugation at 500g during 30 min at room temperature (RT), collected in a centrifuge tube and diluted with 3 volumes of PBS with 2% FCS. Subsequently, the sample was centrifuged at 300g during 5 min at RT and the pellet resuspended with PBS with 2% FCS at a concentration of 10^6^ cells/mL. This cell suspension was processed for flow cytometry.

The sample was centrifuged at maximum speed during 10s, the solution discarded, the pellet resuspended with the residual volume, and incubated with serum AB (20 μl) during 10 min. Then, 20 μl of the following antibodies were added: anti CD34-PerCP, anti CD45-FITC and anti CD133-PE.

The cellular suspension was incubated with antibodies for 20 min in dark at RT. Finally, FACS buffer was added to the sample for cell analysis with the flow cytometer (FACSCalibur). A total of 75,000 events were passed through the flow cytometer.

The number of progenitor cells was determined using specific software (CellQuest). For this analysis we gated a region (R1) in histogram 1 (Figure S1) (CD45/Side Scatter (SSC)) to include all CD45dim to CD45bright events and to exclude debris, platelets, and unlysed erythrocytes, which are CD45 negative. Histogram 2 (SSC vs Forward scatter (FSC)) displays the events that fulfill the criteria of R1. We gated another amorphous polygon region (R2) to exclude erythrocytes but not low forward lymphocytes. Histograms 3 (CD34 vs SSC) and 4 (CD133 vs SSC) display the events that fulfill the criteria of R1+R2. We draw a new region that includes CD133 and CD34 positive cells, respectively, with a low SSC (i.e.R3 and R4). Histogram 5 displays events that fulfill the criteria of all four regions (R1+R2+R3+R4).

**Endothelium-dependent vasodilation (Flow mediated dilation)**

Endothelium-dependent flow-mediated dilation and endothelium-independent nitroglycerine-mediated dilation of the brachial were measured using ultrasound and according to the recommended guidelines. Due of the circadian variations of peripheral vascular tone, the study was performed in all subjects between 8 and 9 am in a quiet, temperature controlled room and after a 12 hour overnight fast. Smokers refrained from smoking in the 12 hours preceding the study.

Endothelial function was studied using high-resolution ultrasound of the brachial artery. A longitudinal section of the right brachial artery was scanned with a vascular probe connected to the ultrasound machine. In order to achieve a steady image throughout the whole study, the probe was fixed with a mechanical clamp, and the sample volume of the pulsed wave Doppler was placed in the middle of the arterial lumen as a reference marker. After a clear image was obtained, a baseline scan was recorded. Endothelium-dependent vasodilation was assessed by analysis of the brachial artery diameter changes in response to an increase in flow. Reactive hyperemia was achieved by the rapid release of a pneumatic pressure cuff placed around the forearm, which was inflated up to 300 mmHg during 4.5 minutes. The pulsed wave Doppler signal of the brachial artery flow was recorded 30 seconds before cuff release and during 1 minute in order to detect the flow increase and during 1-2 minutes more in B mode to detect the maximum vasodilation post-hyperemia, which occurs 55-65 seconds after cuff release. After a 10-15 minutes rest to allow vessel recovery; a second baseline scan was obtained. To assess endothelium-independent vasodilation, 400 μg of sublingual nitroglycerine were administered and a fourth scan was obtained 3 minutes later.

Images were analyzed by an independent observer blinded to the clinical data of the patient. Arterial diameter was measured from two-dimensional echocardiographic images at the peak of the R wave of the ECG with dedicated customized software that includes a 5cm length segment of the brachial artery placing calipers from the trailing edge of the anterior wall interface to the leading edge of the posterior wall interface. Flow-mediated vasodilation (FMD) was used as an index of endothelium-dependent vasodilation and was calculated as the percent change in brachial artery mean diameter after reactive hyperemia over that obtained at baseline. Reactive hyperemia was calculated as the relative ratio of maximal flow after cuff release to that measured at baseline. Vasodilation induced by sublingual nitroglycerine (NTG-VD) was used as an index of endothelium-independent vasodilation and was calculated as the percent change in brachial artery diameter after nitroglycerine administration from the second baseline scan.

**Laboratory analysis**

Venous blood samples were taken for measuring total cholesterol, low and high density lipoproteins, serum creatinine, full blood count, glucose levels, triglyceride and several inflammatory and vascular markers such as interleukin-6 (IL-6), fibrinogen, C-reactive protein (CRP), endothelin-1, nitrites/nitrates, vascular endothelial growth factor (VEGF), angiopoietin-2 and brain natriuretic peptide (BNP).

C-reactive protein (CRP): CRP was measured by a latex-enhanced immunoturbidimetric assay (ADVIA® 24000 Chemistry system)

Nitric oxide (Nox): For measurement of nitric oxide (NO), the release of NO^-^_2_, the stable breakdown product of NO in aqueous medium, was determined by a chemiluminescence detector in an NO analyzer (Sievers Instruments, Inc., Boulder, CO).

Vascular Endothelial Growth Factor (VEGF): The samples of plasma were immunoassayed in duplicate for human vascular endothelial growth factor-A (VEGF-A) using a commercially available quantitative enzyme-linked immunosorbent assay kit that measures VEGF_165_ (Quantikine; R & D Systems, Oxford, England).

Brain natriuretic peptide (BNP) was measured using a fully automated two-site sandwich BNP immunoassay on an Advia Centaur (Siemens Diagnostics, Zurich, Switzerland).

Endothelin (ET): ET was measured by radioimmunoassay (Euro-Diagnostica, Malmö, Sweden) after ET extraction on Sep-Pak C18 cartridges (Waters Associates, Milford, MA). Plasma samples were acidified with 4% acetic acid and applied to cartridges pre-activated with methanol, distilled water and 4% acetic acid. The eluted ET was then concentrated to dryness (Speed Vac concentrator; Savant Instruments Inc., Farmingdale, NY) and reconstituted for radioimmunoassay

Interleukin-6 (IL-6): was measured by ELISA (Biosource, Nivelles, Belgium)

Angiopoietin-2: was measured by ELISA (Quantikine Angiopoietin-2 Immunoassay R&D

Laboratory staff was blinded to clinical data.

**SUPPLEMENTARY FIGURE LEGENDS**

**Figure S1.** Flow cytometric analysis of circulating progenitor cells. Mononuclear cells are gated according to size and granularity on side scatter. Circulating progenitor cells are serially gated with the use of a combination of anti-CD45 anti-CD-133 and anti-CD34 markers. Only a small percentage of cells are positive for the 3 markers. Red dots in histogram 5 are then represented in specific areas on histogram 1, 2, 3 and 4 in order to check the position in the side/forward scatter plot.
